# Reducing the gap of chronic kidney disease in low- and middle-income countries: what is missing?

**DOI:** 10.1016/j.lana.2023.100625

**Published:** 2023-10-31

**Authors:** Darío Sebastián López, Juliana Alexandra Hernández Vargas, Manuel Urina-Jassir, Miguel Urina-Triana, Oscar H. Franco

**Affiliations:** a School of Health and Sport Sciences, MSc Program in Epidemiology, Fundación Universitaria del Área Andina, Bogotá, Colombia; b Department of Global Public Health and Bioethics, Julius Center for Health Sciences and Primary Care, University Medical Center Utrecht, Utrecht, Netherlands; c Department of Medicine, Boston Medical Center/Boston University Chobanian & Avedisian School of Medicine, Boston, MA, United States; d Faculty of Health Sciences, Universidad Simón Bolívar, Barranquilla, Colombia; e Department of Global Public Health and Bioethics, Julius Center for Health Sciences and Primary Care, University Medical Center Utrecht, Utrecht, Netherlands

Chronic kidney disease (CKD) is a major public health problem affecting 9–13% of the world's population.^1^^,^^2^ The fatal consequences of CKD reached more than 2.6 million deaths in 2017.^1^ Most patients living with CKD (≈79%) are diagnosed in advanced stages (3–5), and the awareness of the disease remains low.^1^^,^^3^^,^^4^ These factors greatly impact health systems and patients causing a high economic burden, poor quality of life and disability.^1^^,^^3^ As the population ages and the prevalence of risk factors (hypertension, diabetes, obesity, and cardiovascular disease) rises, the burden of CKD will continue to grow, tripling the current deaths and becoming the 5th leading cause of years of life lost by 2040.^5^

CKD is heterogeneously distributed across regions and countries. Of the total cases, 59% are concentrated in low- and middle-income countries (LMICs), whereas end-stage renal disease (ESRD) is more prevalent in growing economies.^3^^,^^6^ Likewise, these inequalities are also evident when assessing renal replacement therapy (RRT), to which 90% of disadvantaged populations have no access, contributing to higher morbidity and mortality.^6^ Under the current scenario, the estimated number of people needing RRT will increase to 14.5 million by 2030,^6^ and only a third of this population will receive RRT, widening this gap even further over time.^6^ This situation indicates that healthcare systems, especially in LMICs, must strengthen the availability and effective access to treatment for patients with CKD.

On the other hand, preventing CKD and its progression to ESRD is essential. Despite the availability and relatively low cost of CKD risk factors controlling interventions, many countries invest more resources in RRT than primary care strategies for prevention. For example, 2–3% of the healthcare budget in developed countries is spent on treating ESRD, regardless of its low prevalence (0.1–0.2%) in the total population.^3^ In contrast, the prevalence of CKD detection programs is low, ranging from 24% in LMCIs to 32% in high-income countries.^7^ The first step in implementing integrated and cost-effective kidney care is to prioritize investment in the prevention and timely management of CKD, paving the way to sustainable and high-quality care with larger absolute health gains that could also reduce care inequalities.^8^

Many unattended needs from a prevention and screening point of view require urgent intervention, especially in LMICs. Initial efforts must focus on the early detection of CKD, particularly in people at a higher risk, including afro-descendant, those with low access to health care, and those with traditional risk factors (e.g. mainly hypertension and diabetes).^9^ Additionally, lifestyle modifications and pharmacotherapy, have significant benefits and should be the highest priority in LMICs.^8^ Kidney protection therapies include renin-angiotensin-aldosterone system blockers, SGLT2 inhibitors, mineralocorticoid receptor antagonists, and blood pressure, glucose and lipid-lowering treatment, prevention, and control.^3^ Particularly, the recent EMPA-KIDNEY trial showed that empagliflozin therapy lowers the risk of CKD progression or death from cardiovascular causes than placebo,^10^ irrespective of the diagnosis of diabetes. However, tackling prescription barriers and making these medications affordable in LMICs requires consistent and multisectoral strategies.^3^^,^^6^^,^^11^

Despite the different gaps in prevention and care between countries, a common need is establishing a comprehensive framework at national, community, organizational, and patient levels grounded on kidney health awareness. In addition, nationwide and local installed capacity must empower primary care programs and allied healthcare training over the traditional nephrologist-based model that is no longer sustainable, especially in LMICs where the shortage of nephrologists is up to 90%, and primary care ought to play a more primordial role.^11^ Innovative programs to address CKD should focus on region-specific management strategies arising from a deep understanding of sociocultural and demographic differences.^11^ In this context, leadership and governance are crucial to position kidney health as a priority on each country's agenda, particularly in LMICs that lack specific policies.^3^

Lastly, information systems, including clinical and nationwide registries, are fundamental in regulating, planning, and evaluating kidney healthcare. Nevertheless, the availability and scope of CKD registries are heterogeneous. Most of them cover dialysis and transplant populations, whereas a low proportion includes information on non-dialysis CKD care. It is striking that although most countries have renal screening programs for people with hypertension or diabetes, this information is usually missing in surveillance systems.^12^ Comprehensive information systems should also translate data into meaningful and dynamic information to guide policy development, research, and resource allocation.

In conclusion, advancing towards equitable kidney care requires affordable and sustainable policies and strategies tailored to the different challenges in each country and region (Fig. 1). Switching the priorities from an interventionist perspective to a comprehensive framework to strengthen awareness, screening, prevention, and decision-making based on information systems can positively impact clinical and population outcomes, improving the overall health and quality of life of large segments of the population.

**Fig. 1:**
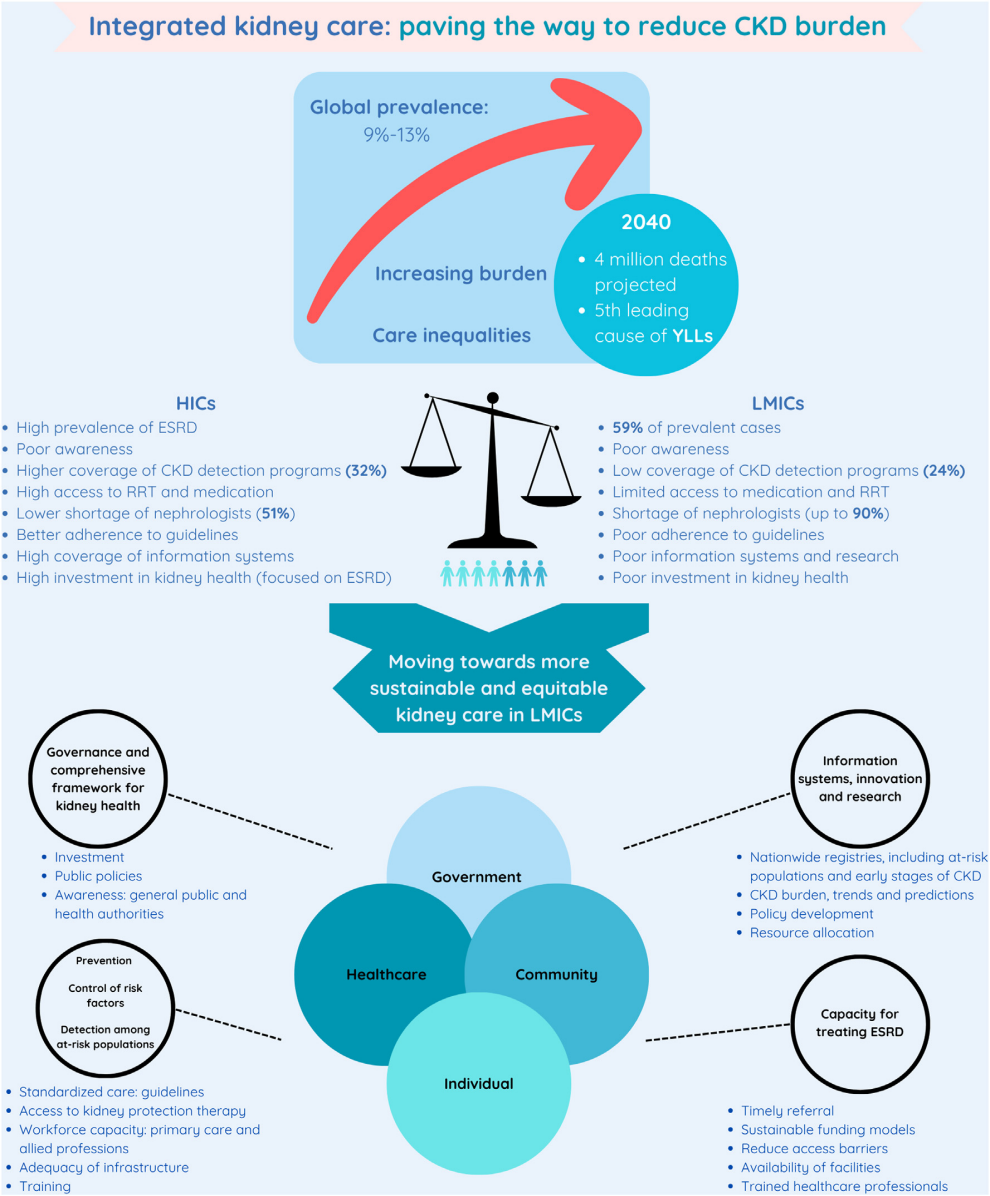
**Challenges and opportunities to improve renal care in low- and middle-income countries**. **Abbreviations**: CKD: chronic kidney disease, ESRD: end-stage renal disease, HICs: high-income countries, LMICs: low- and middle-income countries, RRT: renal replacement therapy, YLLs: years of life lost. **Legend**: The graphical summary depicts the main challenges and opportunities to tackle the growing burden of CKD in LMICs and its associated care inequities. Actions for moving towards sustainable and equitable kidney care models should be based on local and regional needs and capacities. Cohesive efforts of the government, healthcare authorities and providers, community and individuals are the foundations for enhanced prevention, timely assessment, and treatment.

**Source**: Prepared by the authors including information from Bikbov B, et al.,^1^ Bello A, et al.,^3^ Foreman KJ, et al.,^5^ and Stanifer JW, et al.^11^

## Contributors

DSL: conceptualization, writing–original draft, investigation; JAHV: supervision, investigation, and writing–review–editing; MUJ: writing–review and editing; MUT: writing–review and editing; OHF: supervision, writing–review and editing.

## Declaration of interests

We declare no competing interests.
